# Airway protease/antiprotease imbalance in atopic asthmatics contributes to increased Influenza A virus cleavage and replication

**DOI:** 10.1186/1465-9921-13-82

**Published:** 2012-09-19

**Authors:** Matthew J Kesic, Michelle Hernandez, Ilona Jaspers

**Affiliations:** 1 Center for Environmental Medicine, Asthma, and Lung Biology, School of Medicine, University of North Carolina at Chapel Hill, 104 Mason Farm Rd; CB# 7310, Chapel Hill, NC, 27599-7310, USA; 2 The Curriculum in Toxicology, University of North Carolina at Chapel Hill, Chapel Hill, NC, USA; 3 The Department of Pediatrics, School of Medicine, University of North Carolina at Chapel Hill, Chapel Hill, NC, USA; 4 The Department of Microbiology and Immunology, School of Medicine, University of North Carolina at Chapel Hill, Chapel Hill, NC, USA; 5 The Department of Biology, Methodist University, Fayetteville, NC, USA

**Keywords:** Asthma, Influenza A, Protease, Antiprotease, SLPI, TMPRSS2, Hemagglutinin, Susceptibility

## Abstract

Asthmatics are more susceptible to influenza infections, yet mechanisms mediating this enhanced susceptibility are unknown. Influenza virus hemagglutinin (HA) protein binds to sialic acid residues on the host cells. HA requires cleavage to allow fusion of the viral HA with host cell membrane, which is mediated by host trypsin-like serine protease. We show data here demonstrating that the protease:antiprotease ratio is increased in the nasal mucosa of asthmatics and that these changes were associated with increased proteolytic activation of influenza. These data suggest that disruption of the protease balance in asthmatics enhances activation and infection of influenza virus.

## Findings

The viral hemagglutinin (HA) protein expressed on the influenza virion surface is responsible for binding to sialic acid residues on the host cells. Fusion of the virus membrane to the host cell occurs only after HA is proteolytically cleaved, whereas virions with uncleaved HA (HA0) are non-infectious [1,2]. After cleavage by a host trypsin-like serine protease, two protein fragments, HA1 and HA2, are produced. These proteases, in turn, are regulated by mucus antiproteases, such as secretory leukocyte protease inhibitor (SLPI) and α_1_-antitrypsin (A1AT) [2,3]. Our group recently demonstrated that human nasal epithelial cells (NEC) secreted the cellular protease, transmembrane protease serine 2 (TMPRSS2) and the antiprotease, SLPI, and that oxidant-induced disruption of the protease/antiprotease balance, as characterized by increased expression and secretion of TMPRSS2, resulted in increased HA cleavage and viral replication [4].

**Figure 1:**
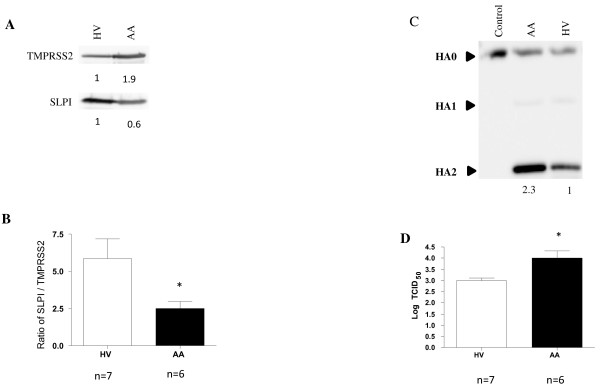
**A) Secreted TMPRSS2 and SLPI.B**) Densitometric analysis to quantitate the amounts of secreted TMPRSS2 and SLPI in all samples. Data were expressed as levels of SLPI: TMPRSS2 ratio for each subject. **C**) Representative immunoblot showing cleavage of viral HA. Densitometry was used to quantitate the amounts of cleaved HA2 protein. Numbers represent relative levels of HA2 in each sample. **D**) Viral titer indicated in log TCID_50_. Unpaired Student’s *t*-test was used for determination of statistically significant differences. Asterisk indicates statistical significance between HVs and AAs; p < 0.05.

Because asthmatics are more susceptible to respiratory viral infections [5] and are thought to be under increased oxidative stress [6], we hypothesize that the disruption in the protease/antiprotease balance in airways results in increased influenza viral cleavage and replication in subjects with allergic asthma (AA). Since the nasal passage is the primary site of infection for several common viruses including influenza, we characterized the protease/antiprotease balance in nasal lavage fluid (NLF) from healthy volunteers (HVs) and AAs. Subject recruitment, sample collection, and analytic techniques are identical to those we have recently reported [4,7]. Demographic data from a total of 13 subjects (7 HVs and 6 AAs) are presented in Table 1. Cell-free NLF from HVs and AAs was used to characterize the differences in protease/antiprotease expression, influenza HA cleavage, and viral replication. By normalizing to total protein concentration present in the cell-free nasal lavage (20μg), immunoblotting revealed that compared to HVs, SLPI expression is decreased and levels of the protease TMPRSS2 are increased, in AAs (Figure 1A). To provide quantitative analysis of this difference, we present our data as an average of the protease/antiprotease balance from 7 HVs and 6AAs. Specifically, densitometric analysis of both TMPRSS2 and SLPI were used to calculate the ratio of SLPI: TMPRSS2 (Figure 1B). These results indicate that AAs display a disruption in the protease/antiprotease balance in the nasal surface liquid, in favor of protease expression. 

**Table 1:** Subject characteristics

	**Healthy volunteer (n=7)**	Atopic asthmatic (n=6)*
**Age (mean years, SD)**	**25.4, 5.7**	**21.5, 2.4**
**Gender (F/M)**	**6/1**	**4/2**
Skin prick test positivity (# of subjects, N)		
Dust Mites	0	4
Trees	0	3
Grasses	0	4
Weeds	0	2
Cat/Dog	0	2
Cockroach	0	2
**Race**	**4 Caucasian**	**4 Caucasian**
	**3 African American**	**1 African American**
		**1 Native Pacific Islander**
BMI (Mean, SD)	26.1, 4.8	23, 2.5

* Atopy was defined by positive epicutaneous skin testing. Asthma status was defined by history, spirometry, and positive methacholine testing. Healthy volunteers were not atopic, nor were they taking any systemic or topical medications. Atopic asthmatics (mild intermittent asthmatics) had to be free of antihistamine use at least 1 week prior to study procedures. They were not taking nasal steroids, inhaled steroids, or leukotriene receptor antagonists.

To determine if the proteases present in the apical surface liquid from nasal lavage are functional and can cleave an intact influenza virion, NLF (50μg of total protein), was incubated with Influenza A/Malaya/302/1954 H1N1 and subsequently analyzed for the various forms of HA using immunoblotting. Figure 1C show NLF from both HVs and AAs was able to cleave HA0 to its products HA1 and HA2, but that NLF from AAs had increased proteolytic activity. Similar to our previous studies [4], we examined whether NLF from AAs and HVs differ in their ability to produce infectious virions using a modified infectivity viral titer assay. These experiments determine whether secreted proteases present in the NLF from HVs and AAs are able to facilitate multiple rounds of viral replication in Madin-Darby canine kidney cells (MDCKs), which require exogenously added protease to become infected and replicate influenza virus [4]. NLF from HVs and AAs was incubated with influenza A/Bangkok/1/79 or with a mock control then added to the MDCK cells for analysis of influenza viral titers. Figure 1D shows that secreted proteases in NLF from healthy volunteers are able to activate influenza virions leading to viral entry and replication in MDCK cells and that these effects are increased in NLF from AAs. Taken together, these results demonstrate that NLF from AAs has a disruption in the secreted protease/antiprotease balance. Using the exact same samples, we also demonstrate that virus incubated with NLF from AA display a significant increase in influenza virus cleavage and replication in MDCK cells.

In conclusion, this is the first study to demonstrate that secreted proteases in NLF from humans can proteolytically activate influenza virions and that these activities are enhanced in AAs. We speculate that disruption of the epithelial protease/antiprotease balance in AAs is a plausible risk-factor for increased susceptibility of these individuals to influenza infection and potentially other viruses such as SARS-CoV and metapneumovirus, which also require proteolytic activation.

## Abbreviations

HA: Hemagglutinin; SLPI: Secretory leukocyte protease inhibitor; A1AT: α_1_-antitrypsin; TMPRSS2: Transmembrane protease serine 2; NLF: Nasal lavage fluid; AA: Allergic asthmatic; NEC: Nasal epithelial cells; HV: Healthy volunteers; MDCK: Madin-Darby canine kidney cells.

## Competing interests

The author(s) declare that they have no competing interests.

## Authors’ contributions

MK designed and carried out the studies, analyzed the data, and drafted the manuscript; MH contributed in the clinical characterization and recruitment of the subject population; IJ contributed to the design, oversaw the coordination of the overall study, and finalized the manuscript. All authors read and approved the final manuscript.

## Funding

The project described was in part supported by National Institutes of Health grant KL2RR025746 (MH), National Institute of Environmental Health Sciences ES013611 (IJ), and Nation Heart, Lung, and Blood Institute HL095163 (IJ).
